# MoS_2_-quantum dot triggered reactive oxygen species generation and depletion: responsible for enhanced chemiluminescence†
†Electronic supplementary information (ESI) available. See DOI: 10.1039/c8sc03511c


**DOI:** 10.1039/c8sc03511c

**Published:** 2018-10-15

**Authors:** Xiangnan Dou, Qiang Zhang, Syed Niaz Ali Shah, Mashooq Khan, Katsumi Uchiyama, Jin-Ming Lin

**Affiliations:** a Beijing Key Laboratory of Microanalytical Methods and Instrumentation , MOE Key Laboratory of Bioorganic Phosphorus Chemistry & Chemical Biology , Department of Chemistry , Tsinghua University , Beijing , 100084 , China . Email: jmlin@mail.tsinghua.edu.cn; b Department of Applied Chemistry , Graduate School of Urban Environmental Sciences , Tokyo Metropolitan University , Minamiohsawa, Hachioji , Tokyo 192-0397 , Japan

## Abstract

Reactive oxygen species (ROS) generation is of intense interest because of its crucial role in many fields. Here we demonstrate that MoS_2_-QDs exhibit a promising capability for the generation of reactive oxygen species, which leads to enhanced chemiluminescence. We discovered that the unique performance is due to hydroxyl radical activation increasing the active catalytic sites on molybdenum sulphide quantum dots (MoS_2_-QDs). The reactive oxygen species, such as hydroxyl radicals (˙OH), superoxide radicals (˙O_2_^–^) and singlet oxygen (^1^O_2_) have been efficiently generated from H_2_O_2_ solution in alkaline conditions. In particular, the maximum ˙OH yield was enhanced significantly (9.18 times) compared to the Fe(ii)/H_2_O_2_ Fenton system under neutral conditions. These findings not only enrich our understanding of the fascinating performance of MoS_2_ QDs, but also provide a new pathway for ROS generation in all kinds of pH environment.

## Abstract 2

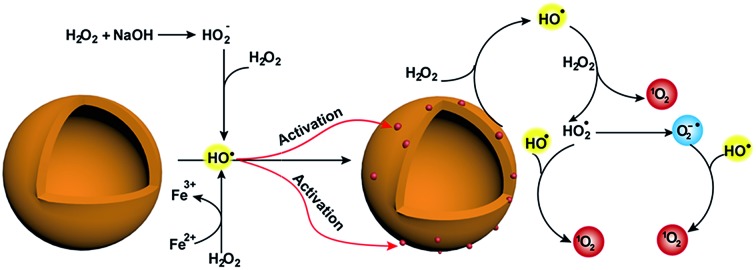
Activated MoS_2_-QDs exhibit a promising capability for the generation of reactive oxygen species.

## Introduction

Reactive oxygen species (ROS) is a collective term for oxygen free radicals and molecules including superoxide radicals (˙O_2_^–^), hydroxyl radicals (˙OH) and singlet oxygen (^1^O_2_), which possess high reactivity compared to molecular O_2_. Generation of abundant ROS is of immense interest in environmental and biological sciences.^1^ To date, the ROS are usually generated using a photocatalytic process *via* light-activated photosensitizers or a chemical process *via* iron-mediated Fenton reaction.^2^ However, the photocatalytic process suffers from undesirable bio-damage by ultra-violet (UV) radiation and low reactive oxygen species production. This has restricted the role of photo-catalysis for biomedical applications.^3^ The Fenton reaction can generate adequate ROS, however, this requires acidic conditions (pH = 3–4). Therefore, alternative reactions and conditions for ROS generation need to be pursued.

Nanoscale molybdenum sulphides (MoS_2_) have gained widespread application in photo-responsive, energy storage and biosensor devices.^4^ Besides, MoS_2_ has been used as an excellent electro-catalyst for the hydrogen evolution reaction (HER).^5^ However, the performance of molybdenum sulphide-quantum dots (MoS_2_-QDs) toward chemiluminescence (CL) based on ROS generation has not been explored.

**Fig. 1:**
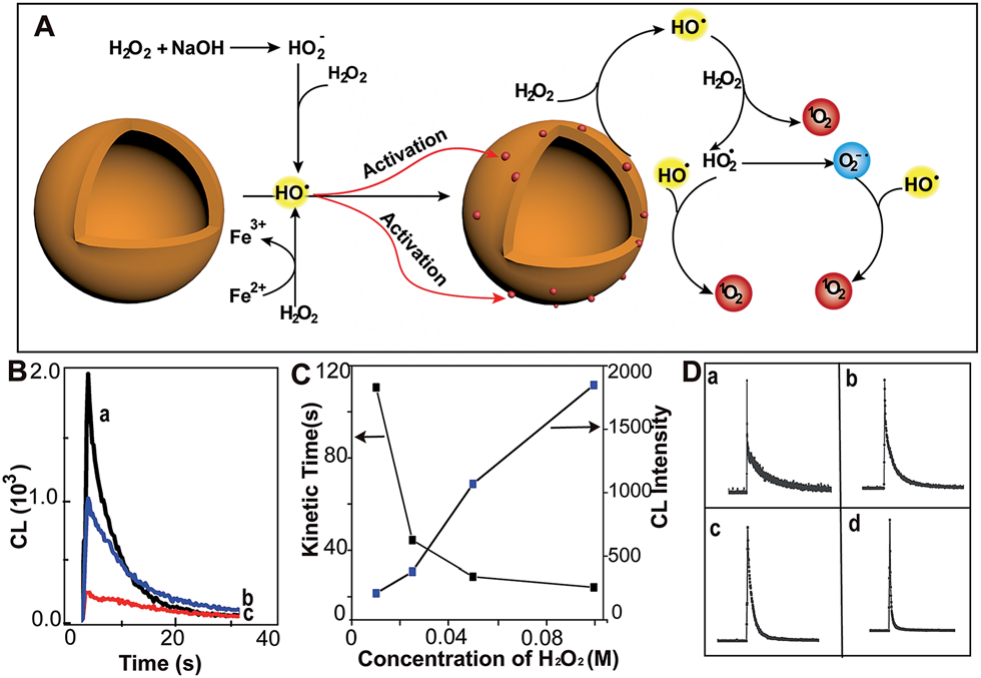
MoS_2_-QDs promote the generation of reactive oxygen species and induce chemiluminescence with H_2_O_2_ in alkaline conditions. (A) Schematic illustration of ROS generation by MoS_2_-QDs. (B) The CL spectrum of injection order with (a) injection of MoS_2_-QDs into a mixture of H_2_O_2_ + NaOH, (b) injection of NaOH into a mixture of H_2_O_2_ + MoS_2_-QDs and (c) injection of H_2_O_2_ into a mixture of MoS_2_-QDs + NaOH. (C) The dose-dependence of H_2_O_2_ concentration on kinetic decay time and CL intensity by injection of MoS_2_-QDs into a H_2_O_2_–NaOH solution. (D) The CL kinetic curve at H_2_O_2_ concentrations of (a) 0.01, (b) 0.025, (c) 0.05, and (d) 0.1 M.

Here, we demonstrate the excellent capability of MoS_2_-QDs to generate ROS from hydrogen peroxide in alkaline solution, which gives rise to CL emission (Fig. 1A). The hydroxyl radicals (˙OH) from intrinsic reactions such as H_2_O_2_ in alkaline medium and the Fe(ii)/H_2_O_2_ Fenton system activate MoS_2_-QDs and generate active catalytic sites on their surface. These active sites then facilitate the conversion of H_2_O_2_ to generate a sufficient amount of ROS, which leads to a strong CL-emission.

## Results and discussion

The MoS_2_-QDs were prepared by solvothermal treatment in *N*,*N*-dimethylformamide (DMF), as proposed by Wang *et al.*^6^ The MoS_2_-QDs exhibited an average size of ∼3.7 nm (Fig. S1, ESI†). The as-prepared product (0.9 mg ml^–1^) was dispersed in water and a transparent pale-yellow solution was obtained, which exhibited a strong fluorescence emission over a wide range of excitation wavelengths (Fig. S2, ESI†).

The chemiluminescence (CL) was produced by MoS_2_-QDs and H_2_O_2_. A stronger CL was obtained with injection of the MoS_2_-QD suspension into a pre-mixed H_2_O_2_/NaOH solution, whereas those with the addition of H_2_O_2_ to NaOH/MoS_2_-QDs or with the addition of NaOH to H_2_O_2_/MoS_2_-QDs, exhibited relatively weaker CL-emission (Fig. 1B). We found that the CL was not only correlated to the injection order, but also to the NaOH concentration. The CL intensity was found to increase with increasing concentration of NaOH and no CL emission was found under acidic or neutral conditions. This was attributed to the remarkable decomposition of H_2_O_2_ in alkaline medium^7^ (80% and 15% at pH = 13 and 10.5 respectively) (Fig. S3, ESI†). The results suggested that the decomposition of H_2_O_2_ plays an important role in the enhanced CL-emission with the addition of MoS_2_-QDs. The hydroxyl radical (˙OH) generation from H_2_O_2_ in the presence of base is given in eqn (1) and (2). Perhydroxyl ions (HO_2_^–^) were formed through the decomposition of H_2_O_2_ in base; then the HO_2_^–^ reacted with undissociated H_2_O_2_ molecules to produce hydroperoxide radicals (HO_2_˙) and ˙OH.^8^

1 H_2_O_2_ + OH^–^ → H_2_O + HO_2_^–^

2 HO_2_^–^ + H_2_O_2_ → HO_2_˙ + ˙OH + OH^–^

Ouyang *et al.* have reported that intrinsic defects can be generated to activate and increase the number of catalytic sites on MoS_2_.^9^ The ˙OH is a strong oxidizing agent, which has a redox potential of 2.8 V,^10^ therefore, the production of ˙OH leads to radical attack on the MoS_2_-QDs and generates defects. This increases the effective catalytic sites of the MoS_2_-QDs and hence facilitates the further decomposition of H_2_O_2_. The occurrence of defects was evidenced by the short lifetime of about 5.2 ns of the MoS_2_-QDs (Fig. S4, ESI†). To clarify the role of ˙OH in the strong CL-emission of the MoS_2_-QD/H_2_O_2_ system, experiments with H_2_O_2_ at different concentrations were performed. We found that the CL-emission intensity of the MoS_2_-QD/H_2_O_2_ system is dependent on the concentration of H_2_O_2_ (Fig. 1C). With increasing [H_2_O_2_] from 0.01 M to 0.1 M, the intensity of CL increased progressively. The CL kinetic decay time was also found to be dependent on the H_2_O_2_ concentration. A flash CL-emission was observed at high H_2_O_2_ concentration (0.1 M), whereas a relatively slower decay was observed at low concentration (Fig. 1D).

**Fig. 2:**
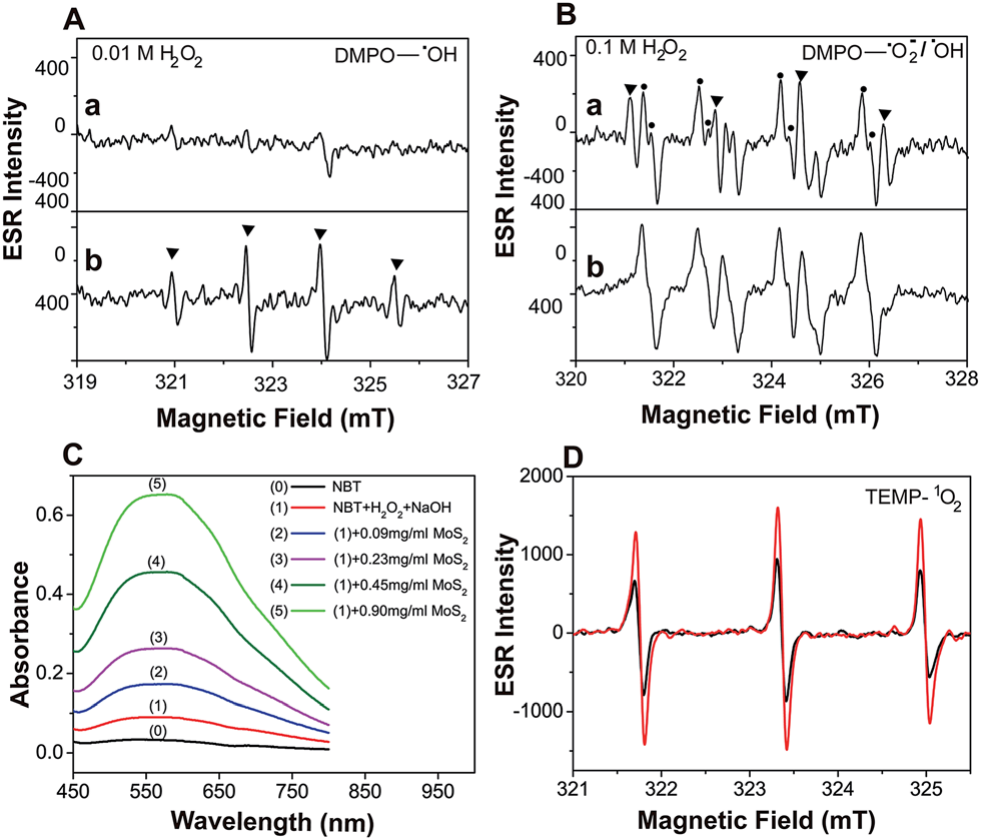
Evaluation of the enhancement of ˙OH, ˙O_2_^–^ and ^1^O_2_ production by MoS_2_-QDs in the H_2_O_2_–NaOH system. (A) ESR spectrum of 5,5-dimethyl-1-pyrroline *N*-oxide (DMPO) with ˙OH obtained from 0.01 M H_2_O_2_ in 0.1 M NaOH (a), and the same as the above conditions but with MoS_2_-QDs (b). (B) ESR spectrum of DMPO with ˙OH or ˙O_2_^–^ obtained from 0.1 M H_2_O_2_ in 0.1 M NaOH (a), and the same as the above conditions but with MoS_2_-QDs (b). (C) The absorbance of NBT in the H_2_O_2_–NaOH system with the addition of MoS_2_-QDs. (D) ESR spectrum of TEMP with ^1^O_2_ production from H_2_O_2_–NaOH (black) and MoS_2_-QDs–H_2_O_2_–NaOH (red).

Electron Spin Resonance (ESR) spectroscopy was performed to directly examine the generation of ˙OH under different H_2_O_2_ conditions. A larger yield of ˙OH was observed as [H_2_O_2_] was increased (Fig. 2A(a) and B(a)). This provides clear evidence that activation of the MoS_2_-QDs is highly dependent on ˙OH formation. No obvious ESR signals for DMPO–OH were observed without MoS_2_-QDs, while the ˙OH was significantly enhanced in the presence of activated MoS_2_-QDs at [H_2_O_2_] = 0.01 M in NaOH (0.1 M) (Fig. 2A(b)). In addition, we found the signal for DMPO–OH and DMPO–˙O_2_^–^ (ref. 11) in the presence of MoS_2_-QDs is stronger than that in the absence of MoS_2_-QDs at H_2_O_2_ = 0.1 M in NaOH (0.1 M) (Fig. 2B(b)). These results suggest the dependence of ˙OH generation on H_2_O_2_ concentration and the ability of the MoS_2_-QDs for ROS generation, which leads to the strong CL-emission of the MoS_2_-QD/H_2_O_2_ system.

Moreover, the UV-vis spectra (Fig. 2C) with nitrotetrazolium blue chloride (NBT) as a superoxide radical (˙O_2_^–^) probe^12^ and ESR spectra (Fig. 2D) with 2,2,6,6-tetramethyl-4-piperidine (TEMP) as a singlet oxygen (^1^O_2_) probe^13^ also showed a significant increase in ˙O_2_^–^ and ^1^O_2_ production in the presence of MoS_2_-QDs. The generation pathways of ˙O_2_^–^ and ^1^O_2_*via* radical reactions are given in eqn (3)–(7).^14^,^15^ We hypothesized that the active sites induced by ˙OH were responsible for the generation of ˙OH, ˙O_2_^–^, and ^1^O_2_ by facilitating the conversion of H_2_O_2_ into more ROS.

3 ˙OH + H_2_O_2_ → HO_2_˙ + H_2_O

4 HO_2_˙ + OH^–^ → ˙O_2_^–^ + H_2_O

5
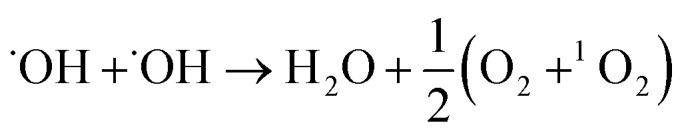

6 ˙OH + HO_2_˙ → H_2_O + O_2_ + ^1^O_2_

7 ˙OH + ˙O_2_^–^ → OH^–^ + ^1^O_2_

**Fig. 3:**
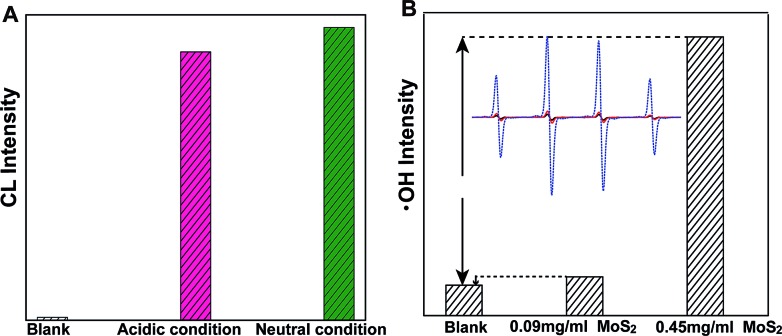
Enhancement of chemiluminescence and hydroxyl radical (˙OH) production by MoS_2_-QDs in the Fe^2+^–H_2_O_2_ system (A) the chemiluminescence of Fe^2+^–H_2_O_2_ (blank); and the CL produced by the addition of MoS_2_-QDs to the Fe^2+^–H_2_O_2_ system under acidic (pH = 3.7) and neutral conditions. (B) The hydroxyl radicals generated from Fe^2+^–H_2_O_2_ (blank) and from the addition of MoS_2_-QDs with a concentrations of 0.09 mg ml^–1^ and 0.45 mg ml^–1^. The inset in panel B is the ESR spectrum of DMPO with ˙OH generated from Fe^2+^–H_2_O_2_ (black) and with the addition of MoS_2_-QDs with concentrations of 0.09 mg ml^–1^ (red) and 0.45 mg ml^–1^ (blue).

In order to further evaluate the dependence of MoS_2_-QD activation and CL-emission on the generation and concentration of ˙OH, a classic ˙OH generation Fenton system was investigated. We found that, MoS_2_-QDs strongly enhanced the CL-emission of the Fe^2+^/H_2_O_2_ system. It was striking that equivalent CL-emission was produced under both neutral and acidic conditions (Fig. 3A). Besides, ˙OH generation was significantly accelerated and compared to the Fe^2+^/H_2_O_2_ system, a 9.18 times greater yield of ˙OH was observed (Fig. 3B). These results confirmed the hypothesis of ROS generation through ˙OH-activated MoS_2_-QDs.

To further verify the generation of ˙OH, the Fenton-oxidation of tetramethylbenzidine (TMB) was introduced, which is known to be oxidized by ˙OH.^16^ In the absence of MoS_2_-QDs, TMB was oxidized slowly and reached a maximum absorbance intensity (*λ*_max_ = 652 nm) after 60 s. Nevertheless, in presence of MoS_2_-QDs, a rapid strong absorbance at 652 nm was observed (Fig. S5, ESI†), indicating a rapid formation of the bluish oxidized TMB because of ˙OH being generated in large amounts. This confirmed that MoS_2_-QD driven Fenton reaction can effectively produce ˙OH.

**Fig. 4:**
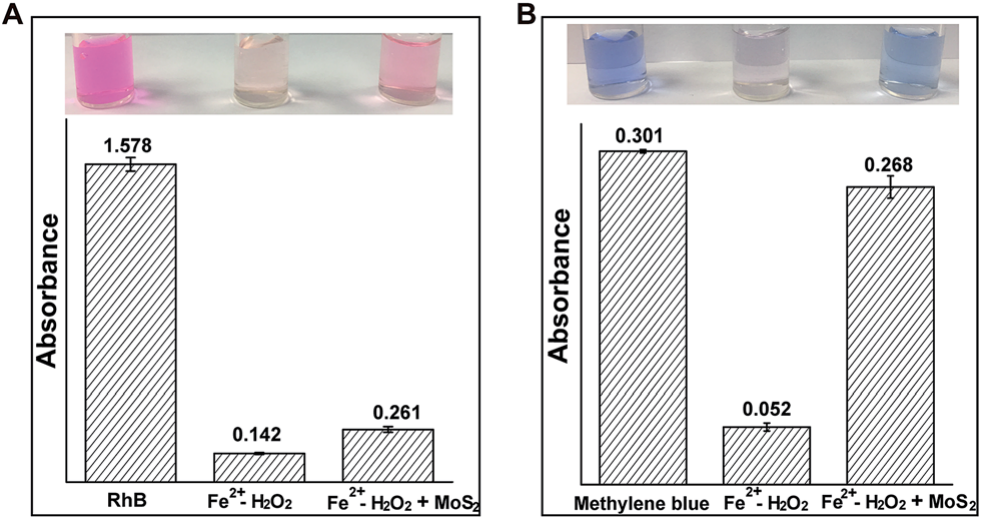
The influence of MoS_2_-QDs on organic contaminant degradation in the Fe^2+^–H_2_O_2_ Fenton system. (A) The absorbance of rhodamine B at 554 nm in the Fe^2+^–H_2_O_2_ and Fe^2+^–H_2_O_2_ + MoS_2_-QD systems; (B) the absorbance of methylene blue at 626 nm in the Fe^2+^–H_2_O_2_ and Fe^2+^–H_2_O_2_ + MoS_2_-QD systems. The solution conditions were 0.01 M H_2_O_2_, 0.45 mg ml^–1^ MoS_2_-QDs, 1 mM Fe^2+^, 5 μg ml^–1^ RhB and 5 μg ml^–1^ MB.

The generation of ˙OH can effectively degrade organic contaminants.^17^ Therefore, phenol, pyrocatechol, rhodamine B (RhB) and methylene blue (MB) were used to find the degradation efficiency and fate of ˙OH in the Fenton (Fe^2+^/H_2_O_2_) and MoS_2_-QD supported Fenton system. Phenolic compound concentrations were measured by using 4-aminoantipyrine for the colorimetric determination.^18^ As expected, a significant decrease of phenol and pyrocatechol (Fig. S6, ESI†) was observed when phenol or pyrocatechol were incubated with the MoS_2_-QD supported Fe^2+^/H_2_O_2_ system as compared to the traditional Fe^2+^/H_2_O_2_ Fenton system, indicating a higher degradation efficiency for phenolic compounds with the Fe^2+^/H_2_O_2_–MoS_2_-QD system. In addition, the degradation of RhB and MB has been detected by the UV-visible method. In the presence of MoS_2_-QDs, MoS_2_-QDs were found to significantly inhibit both RhB and MB degradation efficiency (Fig. 4). Moreover, the presence of MoS_2_-QDs showed a more negative influence on MB degradation, inhibiting the efficiency of MB degradation by 72%, as well as that of RhB degradation efficiency by 8%. This difference towards RhB and MB degradation is probably attributable to the lower reactivity of MB compared to RhB in the Fe^2+^/H_2_O_2_–MoS_2_-QD system, because of significant structural differences between the two different organic fractions (RhB and MB). It has been found previously that ˙OH could be consumed by injection of holes into semiconductor nano-materials.^19^ The selective degradation of phenolic compounds as compared to RhB and MB is probably due to the higher reaction rate of ˙OH with phenolic compounds (*k* ≈ 10^10^ M^–1^ s^–1^),^20^ which makes phenolic compounds more competitive for reacting with ˙OH rather than MoS_2_-QDs in the degradation process. In contrast, ˙OH reacts with MoS_2_-QDs preferentially rather than the aromatic heterocyclic compounds, such as RhB and MB, due to the one order of magnitude lower rate (*k* ≈ 10^9^ M^–1^ s^–1^).^21^ These results strongly indicated that a rapid reaction of MoS_2_-QDs with ˙OH (and ˙O_2_^–^) forming hole-injection ((MoS_2_)˙^+^ QDs) and electron-injection ((MoS_2_)˙^–^ QDs) species (eqn (8) and (9)) occurred.

8 MoS_2_-QDs + ˙OH → (MoS_2_)˙^+^ QDs + OH^–^

9 MoS_2_-QDs + ˙O_2_^–^ → (MoS_2_) ˙^–^ QDs + O_2_

The annihilation of (MoS_2_)˙^+^ and (MoS_2_)˙^–^ resulted in energy release in the form of CL emission,^22^ which has been clarified through a CL-emission spectrum. The CL emission spectrum was centered at 490 nm and 550 nm, which may contain overlap of ^1^O_2_ and MoS_2_ emission^23^ (Fig. S7, ESI†). To verify that this emission was from the recombination of (MoS_2_)˙^+^ and (MoS_2_)˙^–^ and not from the overlap of ^1^O_2_ and MoS_2_-QD emission, the CL-emission was recorded in D_2_O as a solvent instead of H_2_O, because ^1^O_2_ possesses a 13 times longer lifetime in D_2_O than in H_2_O.^24^ Generally, if the CL emission originated from ^1^O_2_, one would expect alteration of the kinetic reaction in D_2_O. However, no obvious change was observed, indicating no contribution of the ^1^O_2_ to the CL-emission due to the annihilation of electron–hole combination of (MoS_2_)˙^+^ QDs and (MoS_2_)˙^–^ QDs (Fig. S8, ESI†).

## Conclusions

For the first time, we have demonstrated the excellent capability of MoS_2_-QDs toward ROS generation. We explored that the synergistic effect of enhanced ROS production and ROS depletion were the main factors leading to the CL-emission of MoS_2_-QDs with H_2_O_2_ in alkaline medium; and with the Fenton reagent under neutral and acidic conditions. These findings present a new pathway for ROS generation over the whole pH-range. Thus, MoS_2_-QDs have potential applications for degradation of organic pollutants and chemo-dynamic therapy.

## Conflicts of interest

There are no conflicts to declare.

## Supplementary Material

Supplementary information
