# Supplementary material for: MoS2-quantum dot triggered reactive oxygen species generation and depletion: responsible for enhanced chemiluminescence
Source: Chem Sci. 2018 Oct 15;10(2):497–500. doi: 10.1039/c8sc03511c (PMC6334262; doi:10.1039/c8sc03511c)
Supplement: Supplementary file 1 [file SC-010-C8SC03511C-s001.pdf]

Electronic Supplementary Material (ESI) for Chemical Science.
This journal is © The Royal Society of Chemistry 2018

Supplementary Information

MoS_2-Quantum Dots Triggered Reactive Oxygen Species Generation and Depletion: Responsible for Enhanced Chemiluminescence

Xiangnan Dou,[ab] Qiang Zhang,[a] Syed Niaz Ali Shah,[a] Mashooq Khan,[a] Katsumi Uchiyama[b] and Jin-Ming Lin*[a]

[a]Beijing Key Laboratory of Microanalytical Methods and Instrumentation, MOE Key Laboratory of Bioorganic Phosphorus Chemistry & Chemical Biology, Department of Chemistry, Tsinghua University, Beijing, 100084, China

[b] Department of Applied Chemistry, Graduate School of Urban Environmental Sciences, Tokyo Metropolitan University, Minamiohsawa, Hachioji, Tokyo 192-0397, Japan

Experimental Section

1. Reagents. All chemicals used in our work were of analytical grade. Hydrogen peroxide (H_2O_2), Sodiu m hydroxide (NaOH), Hydrochloric acid (HCl), Ferrous sulfate($FeSO_4 \cdot 7H_2O$), Rhodamine B(RhB), Methy lene blue (MB)were brought from Beijing Chemical Reagent Co. (Beijing, China). 2,2,6,6-tetramethyl-4-pi peridine (TEMP), 5,5-Dimethyl-1-pyrroline N-oxide (DMPO) and Tetramethylbenzidine (TMB) were purchas ed from J＆K Scientific. Ltd (Beijing, China). Hydrogen peroxide, NaOH and Fe^{2+} solution was prepared freshly before using.

2. Synthesis of MoS_2 QDs. MoS_2 QDs were prepared by top-down method as previously reported. First, 1g of bulk MoS_2 powder was added in 100 mL of N,N-Dimethylformamide (DMF) and kept sonication for 12 h. The solution was put into flask and heated solvothermally at 140℃ for 6 h with vigorous stirring. Afterwards, the resulting mixture was cooled down to ambient temperature naturally and centrifuged for 10 min at 10000 rpm. The MoS_2 QDs was included in light yellow DMF supernatant. To obtain MoS_2 QDs aqueous solution, DMF was removed via rotary evaporation method. Finally, the MoS_2 QDs was dissolved by ultrapure water to a constant volume.

3. Characterization of MoS_2 QDs. The UV-vis spectra were measured by UV-3900 spectrophotometer (Hitachi, Japan). Emission spectra were collected with F-7000 fluorescence spectrophotometer (Hitachi, Japan). The nanoparticle size was recorded by a JEM 2010 electron microscope (JEOL, Japan). Electron paramagnetic resonance (EPR) spectra were measured on a Model JES-FA200 spectrometer (JEOL, Tokyo, Japan). The fluorescence lifetime was recorded by FLSP920 (Edinburgh Instruments, Livingston, UK).

4. Chemiluminescence analysis. Batch CL experiments were carried out with a BPCL luminescence analyzer (Institute of Biophysics, Chinese Academy of Sciences, Beijing, China). The CL spectrum was obtained on the BPCL luminescence analyzer with high-energy cutoff filters from 400 to 640 nm between the flow CL cell and the PMT.

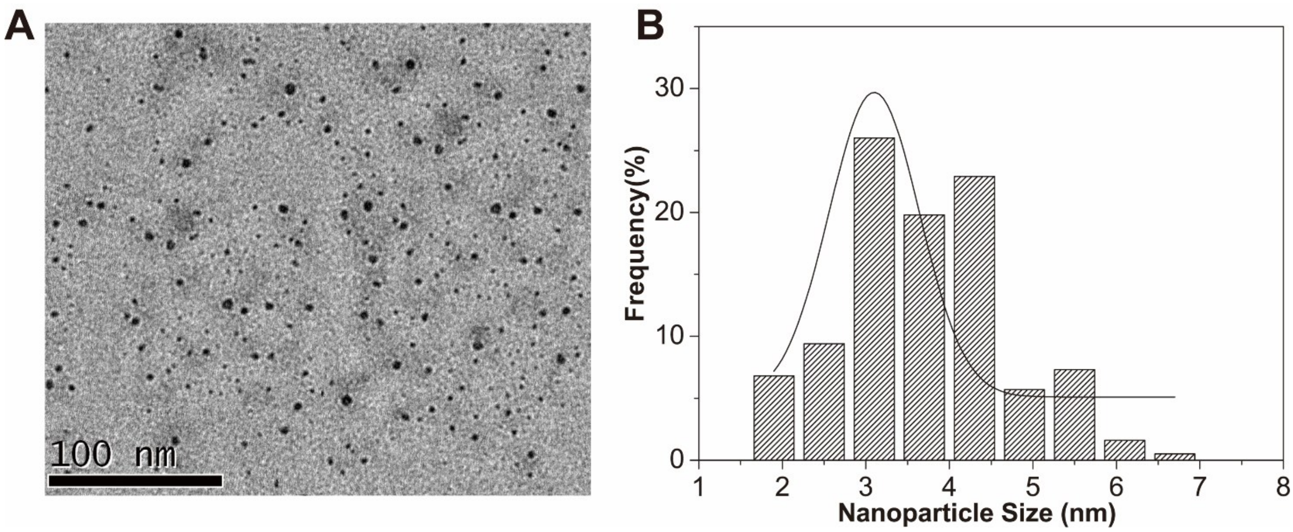

Fig. S1 (A)HRTEM image, and (B) size distribution of MoS_2-QDs.

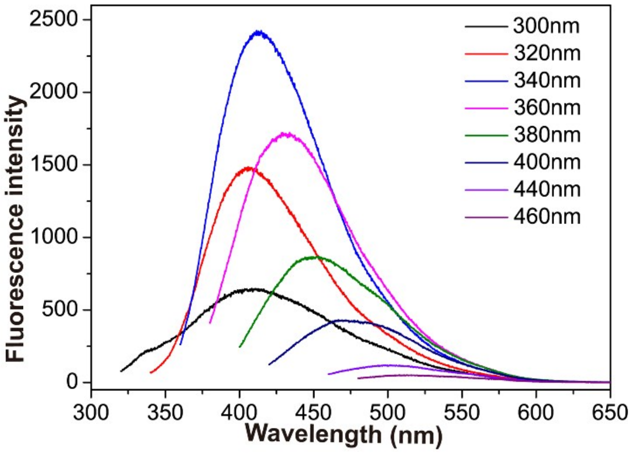

Fig. S2 The fluorescence spectra of MoS_2 QDs (Excitation slit: 2.5 nm, Emission slit: 5 nm; voltage: 700v)

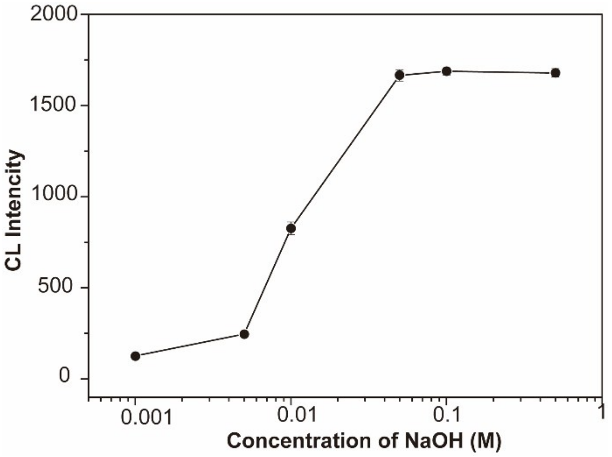

Fig. S3 The correlation of CL intensity with the concentration of sodium hydroxide (NaOH). The solution conditions were 50 μL of 0.1 M H_2O_2, 50 μL of 0.45mg/ml MoS_2 QDs, and 50 μL of sodium hydroxide with concentration of 10^{-3}, 5×10^{-3}, 0.01, 0.05, 0.1, 0.5M respectively. High voltage: 1300 V; interval time was set for 0.1 s.

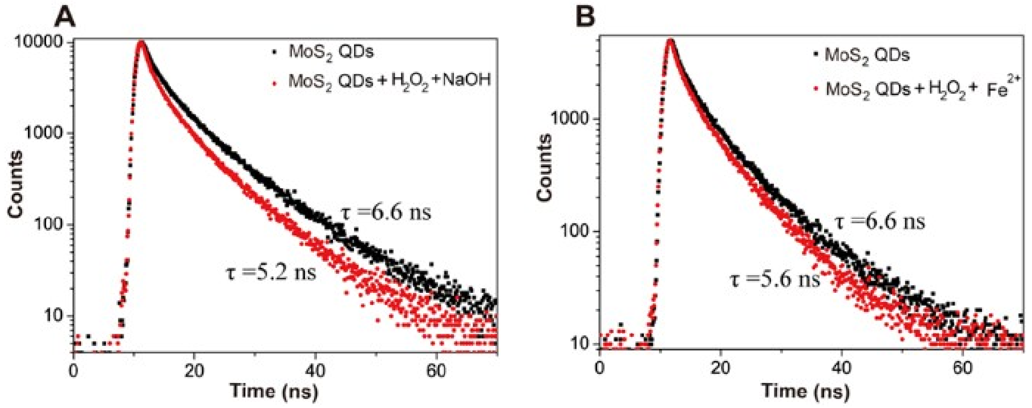

Fig. S4 (A)The fluorescence lifetime decay profile of MoS_2 QDs before (black) and after reaction with H_2O_2-NaOH; (B) The fluorescence lifetime decay curve of MoS_2 QDs before (black) and after reaction with H_2O_2-Fe^{2+}. The solution conditions were 100μl of 0.1 M H_2O_2, 100ul of 0.45mg/ml MoS_2 QDs, 100ul of 0.1 M NaOH and 100μL of 0.1 M Fe^{2+}. The excitation wavelength: 325 nm, emission wavelength: 420 nm.

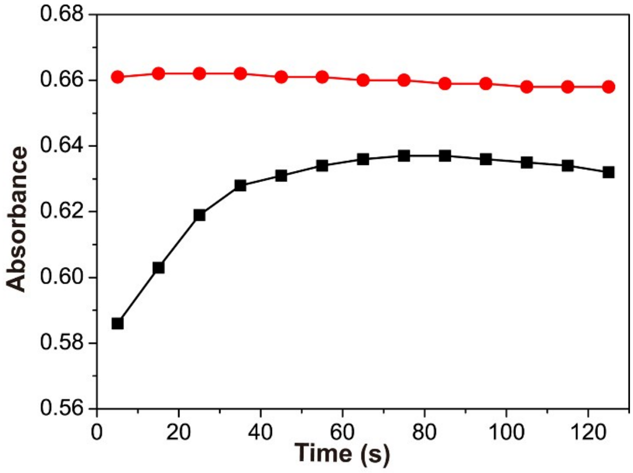

Fig. S5 The absorbance of TMB in H_2O_2-Fe^{2+} system(black) and MoS_2 QDs-H_2O_2-Fe^{2+} system. The solution conditions were 100μL of 0.01 M H_2O_2, 100L of 0.45mg/ml MoS_2 QDs, 100ul of 1mM TMB and 100μL of 1mM Fe^{2+}.

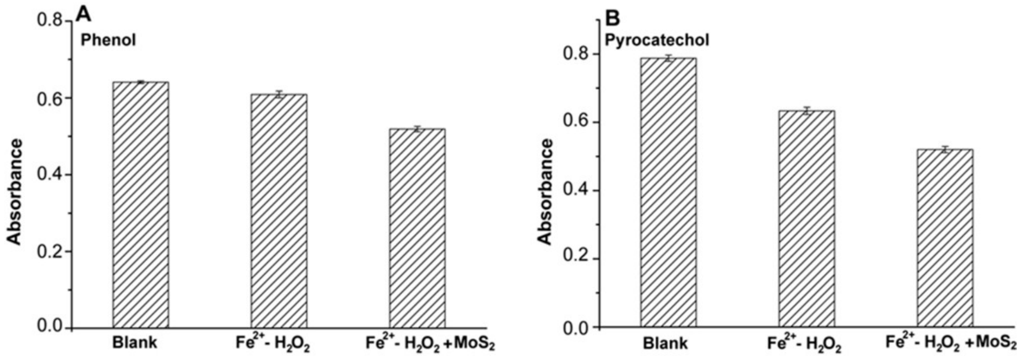

Fig. S6 Comparation of phenolic compounds degradation in Fe^{2+}-H_2O_2 Fenton system and Fe^{2+}-H_2O_2 + MoS_2 QDs system. Phenolic compounds can react with 4-aminoantipyrine forming aminoantipyrine dye with absorbance at wavelength 530.5 nm. The initial concentration of phenolic compounds and final concentration after degradation by Fe^{2+}/H_2O_2 Fenton system and Fe^{2+}/H_2O_2-MoS_2-QDs system were measured by using 4-aminoantipyrine for the colorimetric determination. (A) The absorbance of 4-aminoantipyrine reacted with phenol after incubated in Fe^{2+}-H_2O_2 and Fe^{2+}-H_2O_2 + MoS_2-QDs for 10min; (B) The absorbance of 4-aminoantipyrine reacted with pyrocatechol after incubated in Fe^{2+}-H_2O_2 and Fe^{2+}-H_2O_2 + MoS_2-QDs for 10min. The solution conditions were 0.01 M H_2O_2, 0.45 mg/ml MoS_2-QDs, 1mM Fe^{2+}, 0.01 M $K_3Fe(CN)_6$, 1×10^{-4} M phenol and 5×10^{-4} M pyrocatechol.

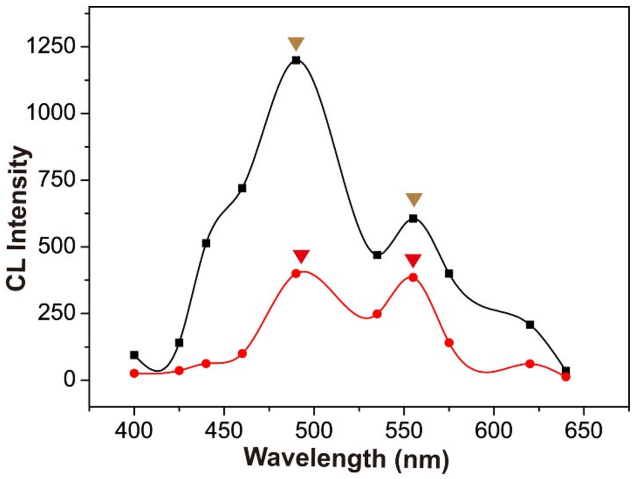

Fig. S7 The CL spectrum of MoS_2 QDs-H_2O_2-NaOH system(red) and MoS_2 QDs-H_2O_2-Fe^{2+} system(black). The solution conditions were 50μL of 0.1 M H_2O_2, 50L of 0.45mg/ml MoS_2 QDs, 50ul of 0.1 M NaOH and 50μL of 0.1 M Fe^{2+}.

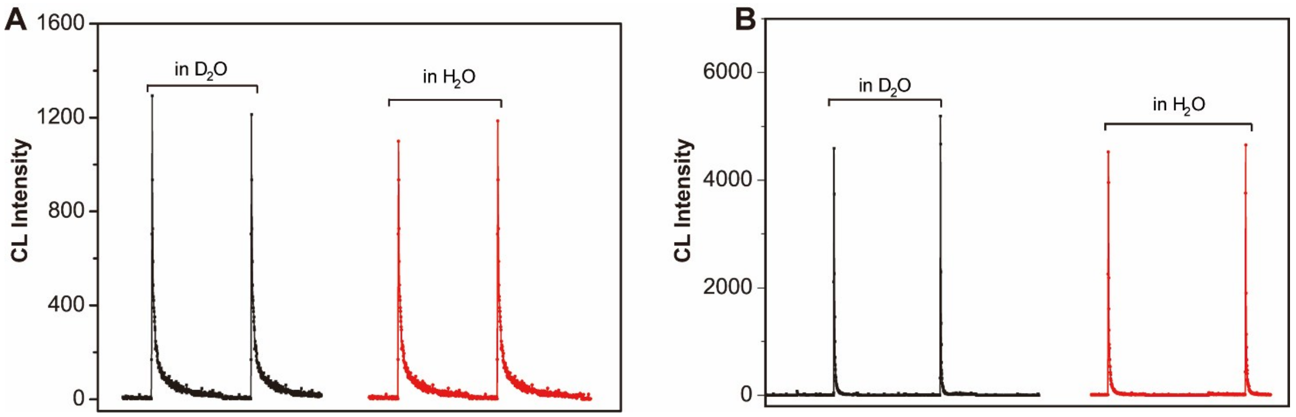

Fig. S8 (A) The comparison of CL of MoS_2 QDs-H_2O_2-NaOH system in D_2O and H_2O reagent respectively. (B) The comparison of CL of MoS_2 QDs-H_2O_2- Fe^{2+} system in D_2O and H_2O reagent respectively.
